# Resveratrol Metabolites Modify Adipokine Expression and Secretion in 3T3-L1 Pre-Adipocytes and Mature Adipocytes

**DOI:** 10.1371/journal.pone.0063918

**Published:** 2013-05-22

**Authors:** Itziar Eseberri, Arrate Lasa, Itziar Churruca, María P. Portillo

**Affiliations:** 1 Nutrition and Obesity Group, Department of Nutrition and Food Science, University of the Basque Country (UPV/EHU), Vitoria, Spain; 2 CIBER de Fisiopatología de la Obesidad y Nutrición (CIBEROBN), Instituto de Salud Carlos III, Madrid, Spain; College of Tropical Agriculture and Human Resources, University of Hawaii, United States of America

## Abstract

**Objective:**

Due to the low bioavailability of resveratrol, determining whether its metabolites exert any beneficial effect is an interesting issue.

**Methods:**

3T3-L1 maturing pre-adipocytes were treated during differentiation with 25 µM of resveratrol or with its metabolites and 3T3-L1 mature adipocytes were treated for 24 hours with 10 µM resveratrol or its metabolites. The gene expression of adiponectin, leptin, visfatin and apelin was assessed by Real Time RT-PCR and their concentration in the incubation medium was quantified by ELISA.

**Results:**

Resveratrol reduced mRNA levels of leptin and increased those of adiponectin. It induced the same changes in leptin secretion. *Trans*-resveratrol-3-*O*-glucuronide and *trans*-resveratrol-4′-*O*-glucuronide increased apelin and visfatin mRNA levels. *Trans*-resveratrol-3-*O*-sulfate reduced leptin mRNA levels and increased those of apelin and visfatin.

**Conclusions:**

The present study shows for the first time that resveratrol metabolites have a regulatory effect on adipokine expression and secretion. Since resveratrol has been reported to reduce body-fat accumulation and to improve insulin sensitivity, and considering that these effects are mediated in part by changes in the analyzed adipokines, it may be proposed that resveratrol metabolites play a part in these beneficial effects of resveratrol.

## Introduction

In recent years, a remarkable range of biological functions has been ascribed to resveratrol. It shows chemopreventive, anti-inflammatory and antioxidant properties [1], [2]. Beneficial cardiovascular effects have also been described [3]. More recently, resveratrol has been proposed as a potential anti-obesity compound [4]-[11], which also improves insulin sensitivity [4], [6], [11], [12].

Several studies have attributed the beneficial effects of resveratrol on body fat accumulation and glycemic control [13]–[16] in part to the changes induced by this polyphenol in adipokines production. Leptin increases energy expenditure and reduces food intake [17], [18]. This adipokine is also related to glycemic control. A physiological increase in plasma leptin levels has been shown to inhibit insulin secretion [19]. Adiponectin increases glucose uptake in muscles and insulin sensitivity, suppresses gluconeogenesis in hepatocytes [20] and increases fatty acid oxidation [21]. With regard to these adipokines, it has been reported that resveratrol reduces leptin expression and secretion and increases adiponectin expression in both *in vitro* and *in vivo* studies [13], [22].

Other adipokines, more recently discovered, such as visfatin and apelin, have been partially involved in obesity and glucose homeostasis. Apelin increases the expression of uncoupling proteins UCP1 and 3, increases energy expenditure and decreases respiratory quotient, resulting in increased fat oxidation [23]. Both of them improve insulin sensitivity and maintain glucose homeostasis in rodents and humans. Visfatin has been shown to play an important role in pancreatic β-cell function by acting as an intra and an extracellular NAD biosynthetic enzyme and regulating glucose-stimulated insulin secretion [24]. Apelin has a close relationship with insulin because it enhances glucose uptake in insulin responsive tissues, such as skeletal muscle [25]–[28]. Derdemezis *et al.* [29] observed decreased visfatin secretion in SGBS adipocytes treated with resveratrol. No data have been reported to date concerning the effect of resveratrol on apelin production.

One of the main concerns of scientists working in the field of resveratrol is its low bioavailability. It has been described that only a small proportion of this molecule reaches plasma and tissues after its oral intake [30], [31]. The concentrations of glucuronide and sulfate metabolites are relatively higher [32]–[34]. In order to increase resveratrol bioavailability, and thus its probable effectiveness as a functional ingredient for prevention and treatment of several diseases, different strategies are under evaluation: combination with other molecules which inhibit its metabolization [35], [36], encapsulation with different excipients (microencapsulation or nanoencapsulation), or searching for more resistant structural analogues (pterostilbene). However, to better determine the target for these research strategies, it is very important to know whether these metabolites exert any effect, and to compare the magnitude of these effects with those of resveratrol.

In this line of research it has been reported that several metabolites show biological activities, such as cancer chemoprotection [37], [38] and anti-inflammation [38], [39]. With regard to the effects of resveratrol metabolites on lipid metabolism there is only one study, reported recently by our group [40].

In this context, the aim of the present study was to determine the effect of resveratrol and the following resveratrol phase II metabolites: *trans*-resveratrol-3-*O*-glucuronide, *trans*-resveratrol-4′-*O*-glucuronide and *trans*-resveratrol-3-*O*-sulfate, on adipokine production in 3T3-L1 maturing pre-adipocytes and mature adipocytes.

## Materials and Methods

### Reagents

Dulbecco’s modified Eagle’s medium (DMEM) was purchased from GIBCO (BRL Life Technologies, Grand Island, NY). *Trans*-Resveratrol (98% purity), *trans*-resveratrol-3-*O*-glucuronide (95% purity), *trans*-resveratrol-4′-*O*-glucuronide (95% purity) and *trans*-resveratrol-3-*O*-sulfate (98% purity) were provided by Bertin Pharma (Montigny le Bretonneux, France).

### Experimental Design

3T3-L1 pre-adipocytes, supplied by American Type Culture Collection (Manassas, VA, USA), were cultured in DMEM containing 10% foetal calf serum (FCS). Two days after confluence (day 0), the cells were stimulated to bring about differentiation with DMEM containing 10% FCS, 10 µg/mL insulin, 0.5 mM isobutylmethylxanthine (IBMX), and 1 µM dexamethasone for 2 days. On day 2, the differentiation medium was replaced by FCS/DMEM medium (10%) containing 0.2 µg/mL insulin. This medium was changed every two days until the cells were harvested. All media contained 1% Penicillin/Streptomycin (10,000 U/mL), and the media for differentiation and maturation contained 1% (v/v) of Biotin and Panthothenic Acid. Cells were maintained at 37°C in a humidified 5% CO_2_ atmosphere.

### Cell Treatment

For the treatment of maturing pre-adipocytes, cells grown in 6-well plates were incubated with either 0.1% ethanol (95%) (control group) or with *trans*-resveratrol, *trans*-resveratrol-3-*O*-glucuronide, *trans*-resveratrol-4′-*O*-glucuronide or *trans*-resveratrol-3-*O*-sulfate, at 25 µM (diluted in 95% ethanol) during differentiation. Media containing resveratrol or its metabolites were changed every two days: on day 0, day 2, day 4 and day 6. On day 8, supernatant was collected for adipokine determination in the media and cells were used for RNA extraction and gene expression measurement. Each experiment was performed 3 times.

For the treatment of mature adipocytes, cells grown in 6-well plates were incubated with the same molecules, at 10 µM (diluted in 95% ethanol) on day 12 after differentiation because at that day >90% of cells were mature adipocytes with visible lipid droplets. After 24 hours, supernatant was collected for adipokine determination in the media and cells were used for RNA extraction and gene expression measurement. Each experiment was performed 3 times.

The reason for using different doses in maturing pre-adipocytes and mature adipocytes was based on the results obtained in the same sets of cells when the delipidating effect of resveratrol metabolites was assessed. In this previous experiment, we analyzed the effects of different doses (1, 10 and 25 µM) and it was observed that in the case of maturing pre-adipocytes the effective dose for reducing triacylglycerol content was 25 µM, and in the case of mature adipocytes this was 10 µM (even though resveratrol also reduced triacylglycerol content at 1 µM) [40].

### Adipokine Concentration in the Media

Commercial kits were used to assess concentrations of apelin, visfatin, leptin and adiponectin in the media by ELISA (EK-003-80, Phoenix Europe GMBH, Karlsruhe, Germany and RAG004R, RD191001100 and RD293023100R, Biovendor, Brno, Czech Republik, respectively).

### Extraction and Analysis of RNA and Quantification by Reverse Transcription-polymerase Chain Reaction (Real Time RT-PCR)

RNA samples were extracted from cells by using Trizol (Invitrogen, Carlsbad, CA, USA), according to the manufacturer’s instructions. The integrity of the RNA extracted from all samples was verified and quantified using a RNA 6000 Nano Assay (Thermo Scientific, Wilmington, DE, USA). RNA samples were then treated with DNase I kit (Applied Biosystems, Foster City, CA, USA) to remove any contamination with genomic DNA.

One µg of total RNA in a total reaction volume of 20 µL was reverse transcribed using the iScript cDNA Archive Kit (Applied Biosystems Inc., Foster City, CA, USA) according to the manufacturer’s protocols. Reactions were incubated initially at 25°C for 10 min and subsequently at 37°C for 120 min and 85°C for 5 min.

Relative leptin, adiponectin, visfatin and apelin mRNA levels were quantified using Real-Time PCR with an iCycler™ - MyiQ™ Real Time PCR Detection System (BioRad, Hercules, CA, USA). β-actin mRNA levels were similarly measured and served as the reference gene. The PCR reagent mixture consisted of 1 µL of each cDNA (10 pmol/µL), SYBR® Green Master Mix (Applied Biosystems, Foster City, CA, USA) and the upstream and downstream primers (300nM each) were used for leptin and adiponectin. In the case of visfatin and apelin, the reagent mixture consisted of 1 µL of each cDNA, Premix Ex TaqTM (Takara, USA) and the upstream and downstream primers (600 nM for visfatin and apelin; 300nM for β-actin) and probe (1 µM visfatin; 0.5 µM apelin and β-actin). Specific primers and probes were synthesized commercially (Eurogentec, Liège, Belgium) (Table 1).

**Table 1:** Primers for PCR amplification of each studied gene.

	Sense primer	Antisense primer	Probe
SYBR® Green RT-PCR:			
Leptin	5′- TGG ACC AGA CTC TGGCAG TC -3′	5′- AGG ACA CCA TCCAGG CTC TC -3′	
Adiponectin	5′-TG TAG GAT TGT CAGTGG ATC TG-3′	5′-GCT CTT CAG TTGTAG TAA CGT CAT C-3′	
β-Actin	5′- ACG AGG CCC AGA GCA AGA -3′	5′- GGT GTG GTG CCA GAT CTT CTC -3′	
TaqMan RT-PCR:			
Visfatin	5′- CCG GCC CGA GAT GAA TGC -3′	5′- GGA ATA AAC TTT GCT TGT GTT GGG -3′	5′-FAM- AGC CGA GTT CAA CAT CCT GCTGGC -TAMRA- 3′
Apelin	5′- ATT TAA GGA CACGCT GAT CAA AGG-3′	5′- AGT CCC GAA AGT ATT CAAAAG CAG-3′	5′- AAA CAG AAG GCA CCC ACCAGG GCT-3′
β-Actin	5′- TCT ATG AGG GCT ACG CTC TCC -3′	5′- CAC GCT CGG TCA GGATCT TC -3′	5′- FAM- CCT GCG TCT GGA CCT GGCTGG C -TAMRA -3′

PCR parameters were as follows: initial 2 min at 50°C, denaturation at 95°C for 10 min followed by 40 cycles of denaturation at 95°C for 30s, annealing at 60°C for 30 s, and extension at 60°C for 30 s. All sample mRNA levels were normalized to the values of β-actin and the results expressed as fold changes of threshold cycle (Ct) value relative to controls using the 2^−ΔΔCt^ method [41].

### Statistical Analysis

Results are presented as mean±standard error of the mean. Statistical analysis was performed using SPSS 19.0 (SPSS Inc. Chicago, IL, USA). Comparisons between each treatment with the control were analyzed by Student’s *t* test. Statistical significance was set-up at the *P*<0.05 level.

## Results

### Effects of Resveratrol and its Metabolites on Adiponectin Expression

**Figure 1:**
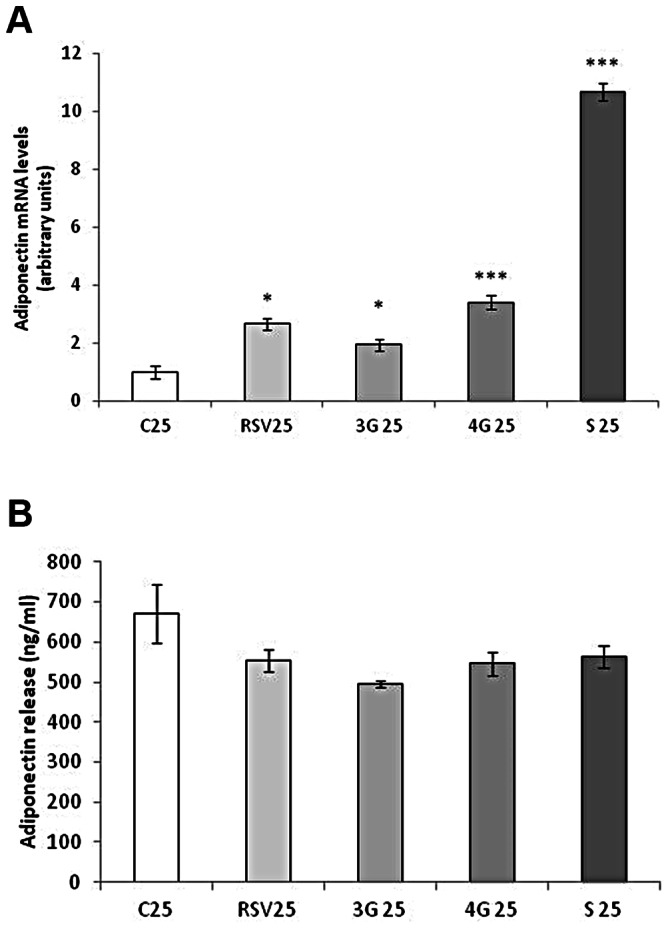
Effects of 25 µM *trans*-resveratrol (RSV), *trans*-resveratrol-3-*O*-glucuronide (3G), *trans*-resveratrol-4′-*O*-glucuronide (4G) or *trans*-resveratrol-3-*O*-sulfate (S) on the mRNA expression (A) and protein concentration in the culture media (B) of adiponectin in 3T3-L1 maturing pre-adipocytes treated from day 0 to day 8 of differentiation. Values are means±SEM. Comparisons between each treatment with the control were analyzed by Student’s *t* test. The asterisks represent differences *vs.* the control (*P<0.05, ***P<0.001).

**Figure 2:**
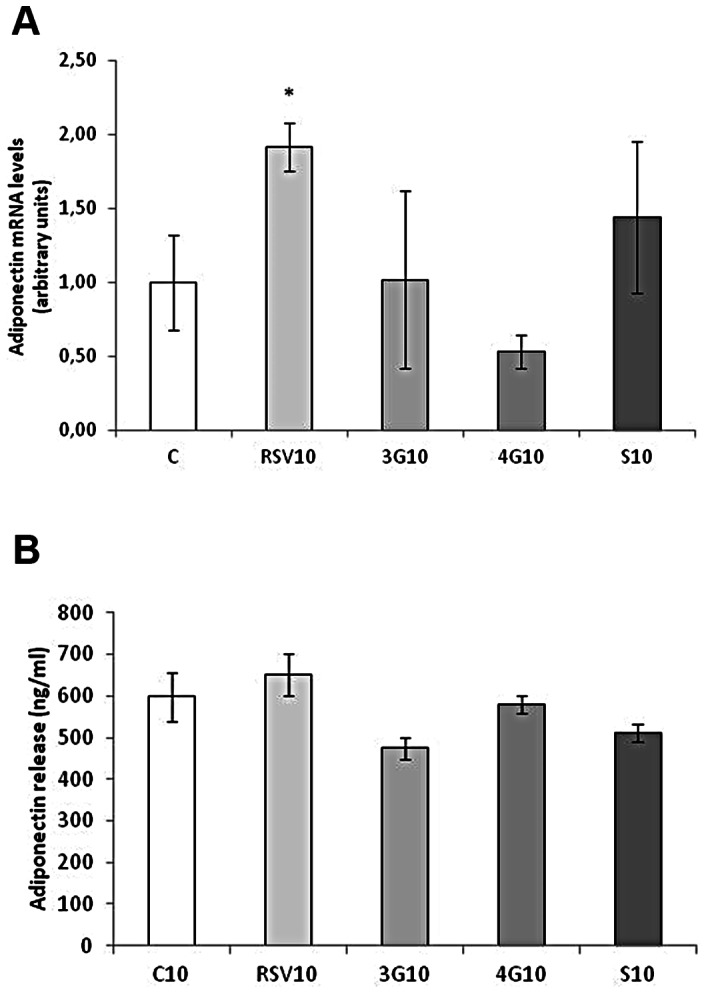
Effects of 10 µM *trans*-resveratrol (RSV), *trans*-resveratrol-3-*O*-glucuronide (3G), *trans*-resveratrol-4′-*O*-glucuronide (4G) or *trans*-resveratrol-3-*O*-sulfate (S) on the mRNA expression (A) and protein concentration in the culture media (B) of adiponectin in 3T3-L1 mature adipocytes treated for 24 hours on day 12 of differentiation. Values are means±SEM. Comparisons between each treatment with the control were analyzed by Student’s *t* test. The asterisks represent differences *vs.* the control (*P<0.05).

After treating maturing pre-adipocytes from day 0 to day 8, resveratrol and the three analyzed metabolites increased adiponectin expression (Figure 1A). Nevertheless, no changes were observed in the secretion of this adipokine (Figure 1B). In mature adipocytes, only resveratrol increased adiponectin expression but no differences were observed in the concentration in the medium after 24 hours of treatment (Figure 2A and 2B).

### Effects of Resveratrol and its Metabolites on Leptin Expression

**Figure 3:**
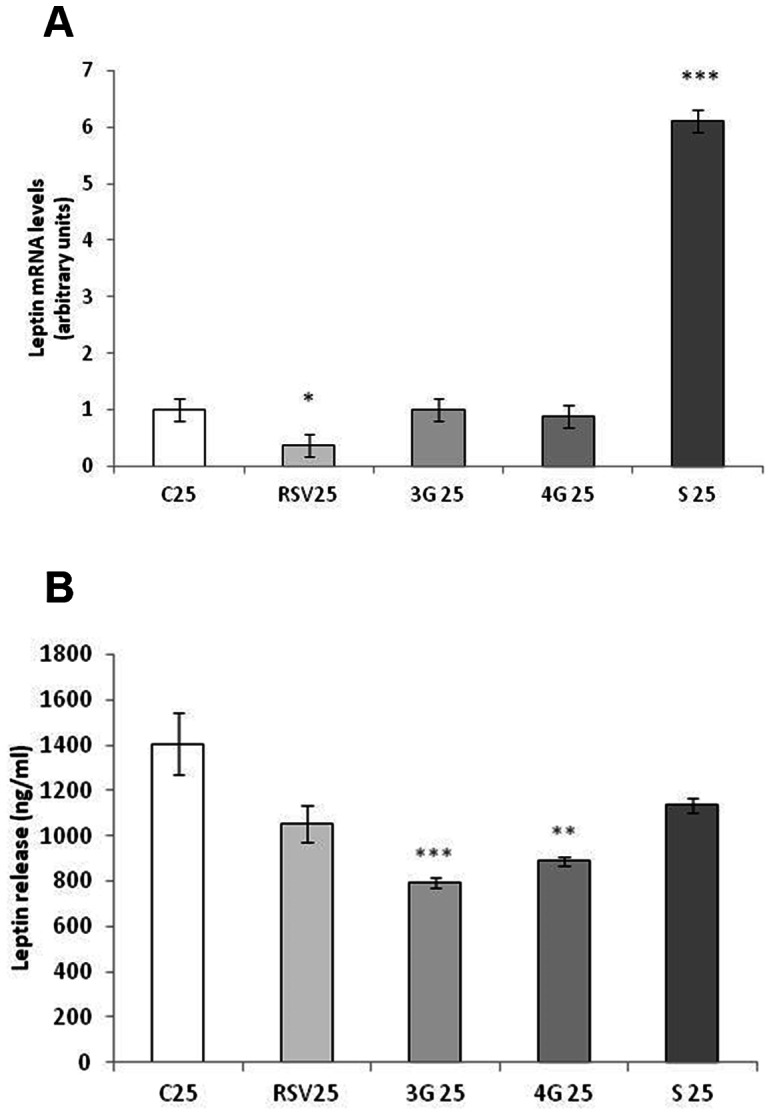
Effects of 25 µM *trans*-resveratrol (RSV), *trans*-resveratrol-3-*O*-glucuronide (3G), *trans*-resveratrol-4′-*O*-glucuronide (4G) or *trans*-resveratrol-3-*O*-sulfate (S) on the mRNA expression (A) and protein concentration in the culture media (B) of leptin in 3T3-L1 maturing pre-adipocytes treated from day 0 to day 8 of differentiation. Values are means±SEM. Comparisons between each treatment with the control were analyzed by Student’s *t* test. The asterisks represent differences *vs.* the control (*P<0.05, **P<0.01, ***P<0.001).

**Figure 4:**
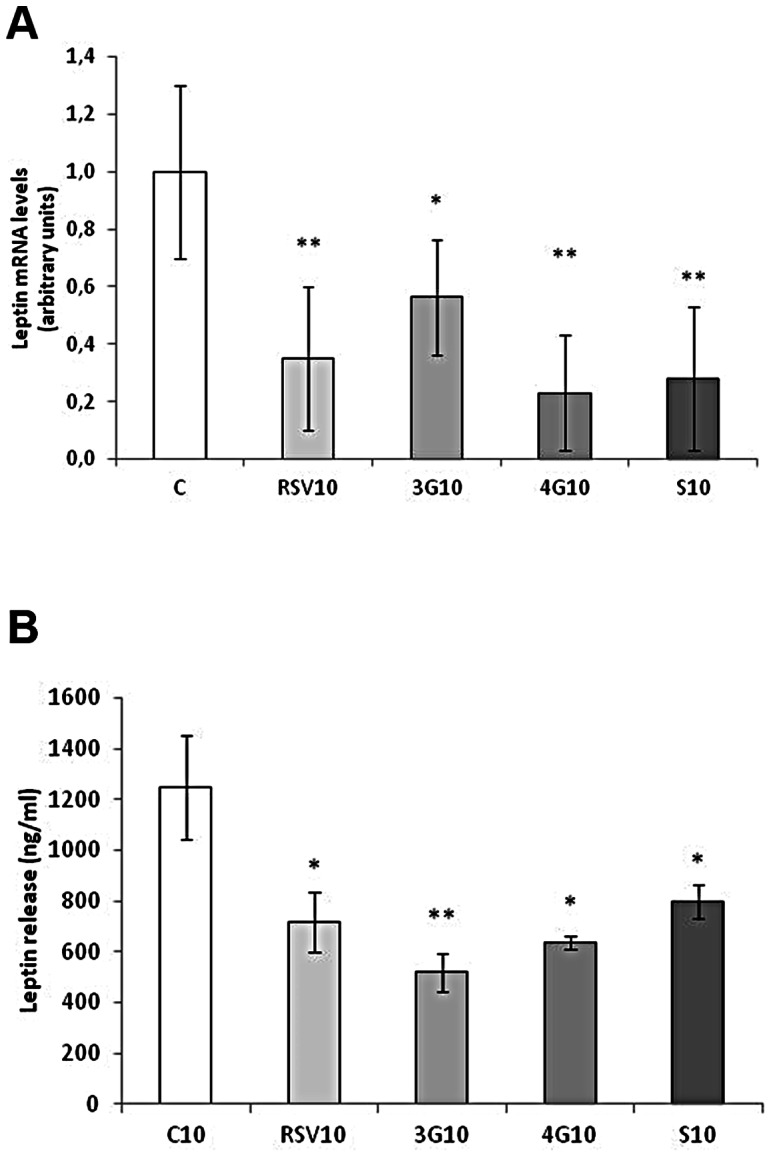
Effects of 10 µM *trans*-resveratrol (RSV), *trans*-resveratrol-3-*O*-glucuronide (3G), *trans*-resveratrol-4′-*O*-glucuronide (4G) or *trans*-resveratrol-3-*O*-sulfate (S) on the mRNA expression (A) and protein concentration in the culture media (B) of leptin in 3T3-L1 mature adipocytes treated for 24 hours on day 12 of differentiation. Values are means±SEM. Comparisons between each treatment with the control were analyzed by Student’s *t* test. The asterisks represent differences *vs.* the control (*P<0.05, **P<0.01).

As far as maturing pre-adipocytes are concerned, resveratrol reduced, while *trans*-resveratrol-3-*O*-sulfate increased, leptin mRNA levels No changes were induced by glucuronide metabolites (Figure 3A). However, glucuronide metabolites reduced leptin secretion to the media, and resveratrol and *trans*-resveratrol-3-*O*-sulfate showed a tendency towards reduced values (Figure 3B). With regard to mature adipocytes, both the expression and secretion of leptin were reduced by resveratrol and by all the metabolites analyzed (Figure 4A and 4B).

### Effects of Resveratrol and its Metabolites on Visfatin Expression

**Figure 5:**
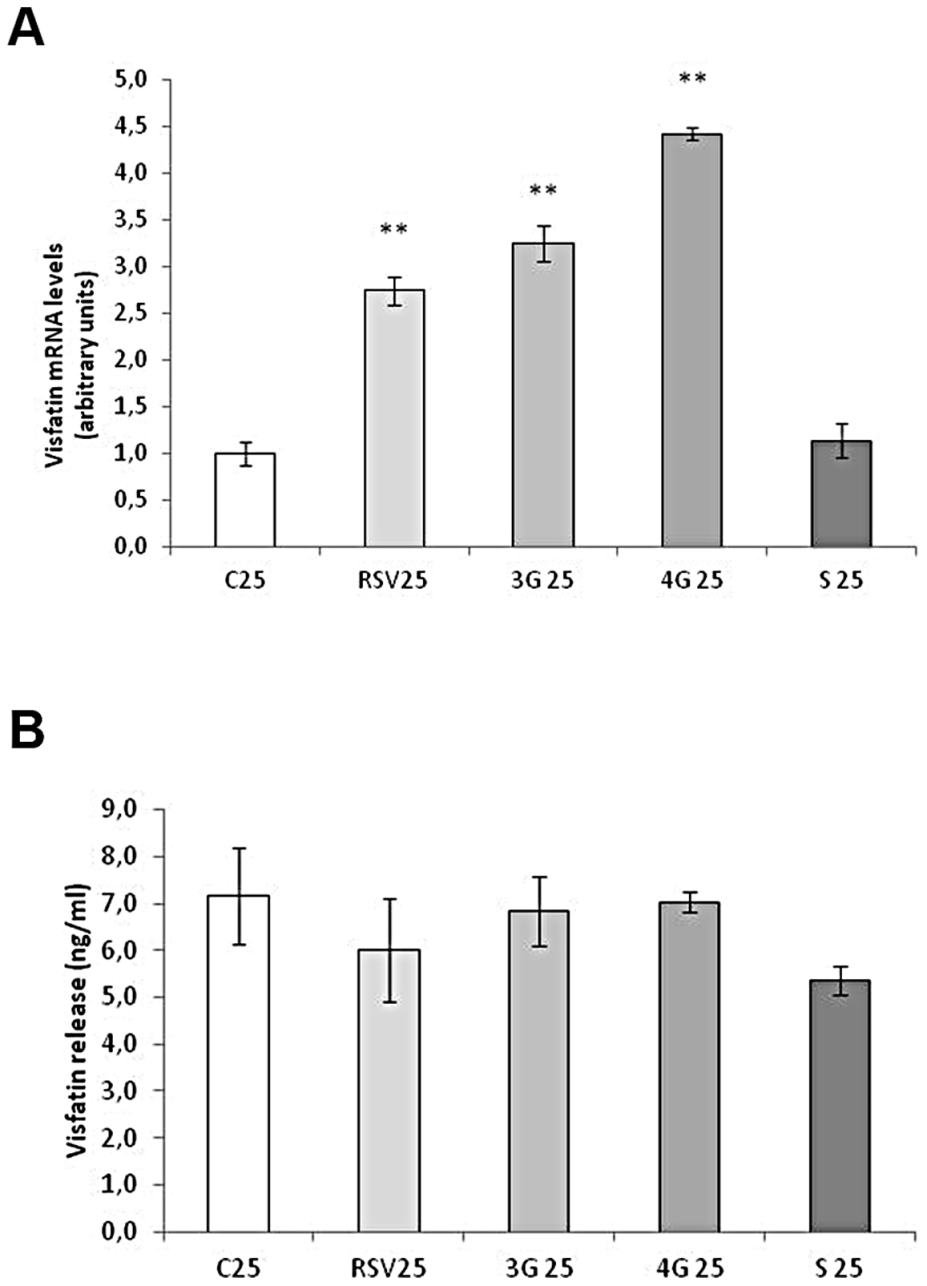
Effects of 25 µM *trans*-resveratrol (RSV), *trans*-resveratrol-3-*O*-glucuronide (3G), *trans*-resveratrol-4′-*O*-glucuronide (4G) or *trans*-resveratrol-3-*O*-sulfate (S) on the mRNA expression (A) and protein concentration in the culture media (B) of visfatin in 3T3-L1 maturing pre-adipocytes treated from day 0 to day 8 of differentiation. Values are means±SEM. Comparisons between each treatment with the control were analyzed by Student’s *t* test. The asterisks represent differences *vs.* the control (**P<0.01).

**Figure 6:**
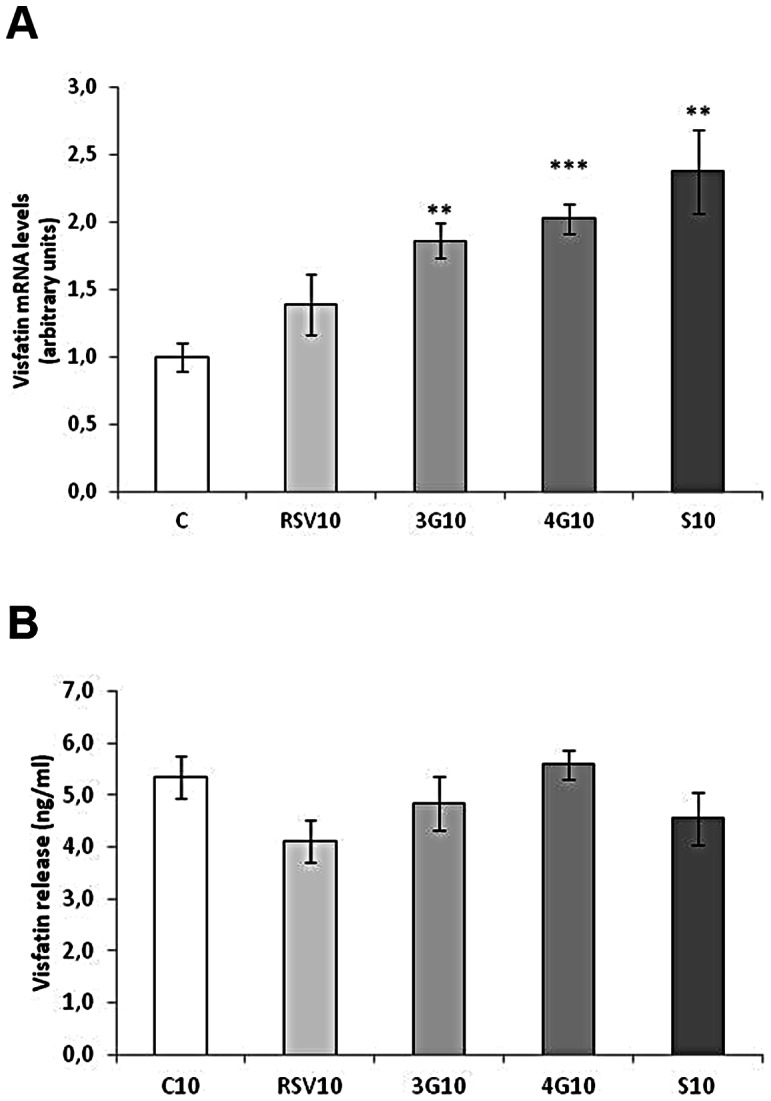
Effects of 10 µM *trans*-resveratrol (RSV), *trans*-resveratrol-3-*O*-glucuronide (3G), *trans*-resveratrol-4′-*O*-glucuronide (4G) or *trans*-resveratrol-3-*O*-sulfate (S) on the mRNA expression (A) and protein concentration in the culture media (B) of visfatin in 3T3-L1 mature adipocytes treated for 24 hours on day 12 of differentiation. Values are means±SEM. Comparisons between each treatment with the control were analyzed by Student’s *t* test. The asterisks represent differences *vs.* the control (**P<0.01, ***P<0.001).

Resveratrol and the glucuronide metabolites increased visfatin expression in maturing pre-adipocytes, without changes in protein secretion. *Trans*-resveratrol-3-*O*-sulfate did not induce any change (Figure 5A). No changes were observed in visfatin secretion (Figure 5B). In mature adipocytes, while resveratrol did not affect the mRNA expression of this adipokine, the three metabolites increased its mRNA levels (Figure 6A). Despite this increase, visfatin concentration in the media did not change (Figure 6B).

### Effects of Resveratrol and its Metabolites on Apelin Expression

**Figure 7:**
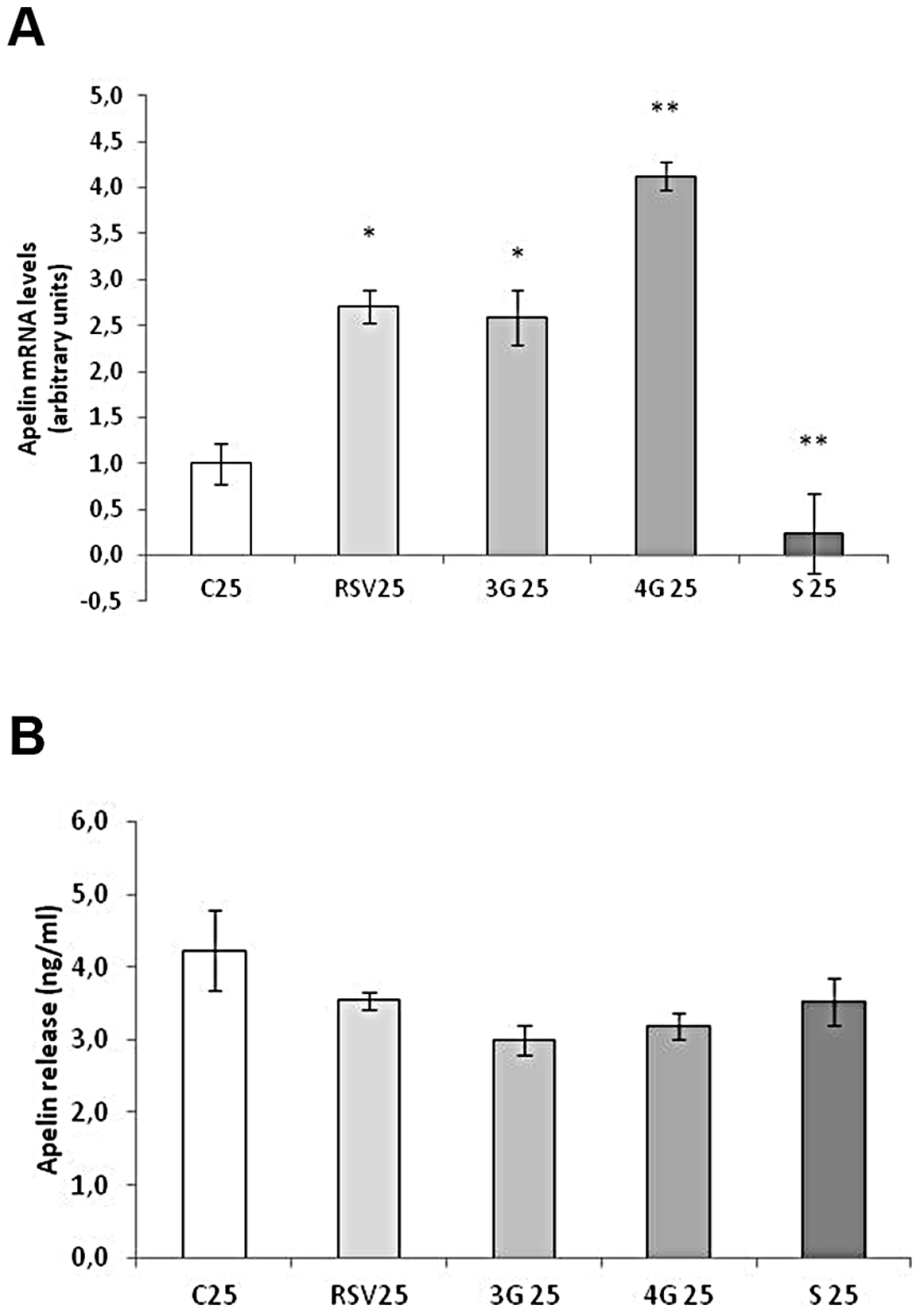
Effects of 25 µM *trans*-resveratrol (RSV), *trans*-resveratrol-3-*O*-glucuronide (3G), *trans*-resveratrol-4′-*O*-glucuronide (4G) or *trans*-resveratrol-3-*O*-sulfate (S) on the mRNA expression (A) and protein concentration in the culture media (B) of apelin in 3T3-L1 maturing pre-adipocytes treated from day 0 to day 8 of differentiation. Values are means±SEM. Comparisons between each treatment with the control were analyzed by Student’s *t* test. The asterisks represent differences *vs.* the control (*P<0.05, **P<0.01).

**Figure 8:**
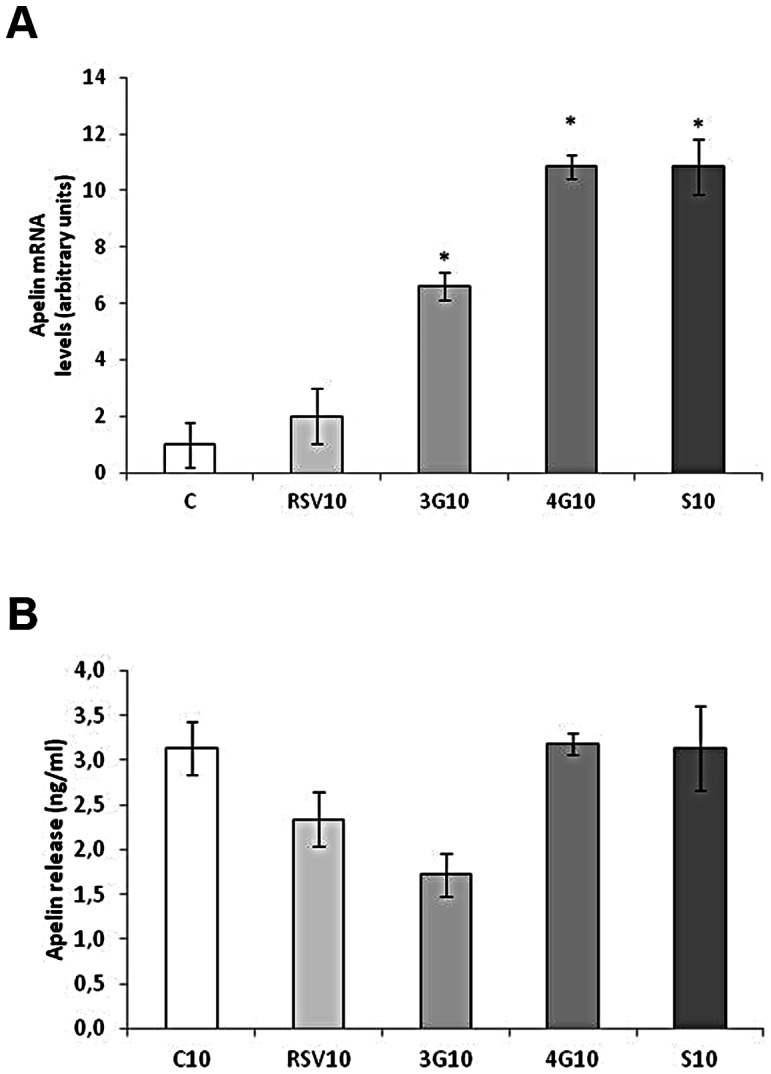
Effects of 10 µM *trans*-resveratrol (RSV), *trans*-resveratrol-3-*O*-glucuronide (3G), *trans*-resveratrol-4′-*O*-glucuronide (4G) or *trans*-resveratrol-3-*O*-sulfate (S) on the mRNA expression (A) and protein concentration (B) of apelin in 3T3-L1 mature adipocytes treated for 24 hours on day 12 of differentiation. Values are means±SEM. Comparisons between each treatment with the control were analyzed by Student’s *t* test. The asterisks represent differences *vs.* the control (*P<0.05).

Resveratrol and glucoronide metabolites increased apelin mRNA levels in maturing pre-adipocytes, whereas *trans*-resveratrol-3-*O*-sulfate reduced them (Figure 7A). Nevertheless, no changes were observed in apelin secretion (Figure 7B). In mature cells, only the metabolites increased apelin expression without changing its secretion (Figure 8A and 8B).

## Discussion

Following ingestion, most resveratrol metabolizes rapidly, resulting in up to 20-fold higher concentrations of circulating conjugates, and less than 1% of the parent compound [32], [42]. Thus, it is important to know whether resveratrol metabolites show biological activities and if as a result they contribute to the beneficial effects of resveratrol on health. Some authors have demonstrated the anticarcinogenic effect of resveratrol metabolites [38], [43].

In this context, our group had previously reported the delipidating effect of resveratrol metabolites, by using the same sets of cells as those used in the present study [40]. Specifically, *trans*-resveratrol-4′-*O*-glucuronide and *trans*-resveratrol-3-*O*-sulfate induced similar delipidating effects to that of resveratrol in maturing pre-adipocytes; *trans*-resveratrol-3-*O*-glucuronide and *trans*-resveratrol-4′-*O*-glucuronide showed an important delipidating effect (although lower than that of resveratrol) in mature adipocytes. Consequently, we suggested that both resveratrol and resveratrol metabolites were involved, to greater or lesser extent, in the anti-obesity effect of this polyphenol. We differentiated the effects of resveratrol metabolites on maturing pre-adipocytes and mature adipocytes because these two cell types play different roles in obesity development. Thus, during childhood and adolescence, obesity is mainly induced by hyperplasia, which implies the differentiation of pre-adipocytes into mature adipocytes. By contrast, in adulthood, obesity is mainly induced by hypertrophy of mature adipocytes.

Several positive effects of resveratrol are mediated by changes in adipokine production. Adipokines produced by adipose tissue can act in an autocrine or in a paracrine manner to modify metabolic pathways involved in lipid metabolism and glucose homeostasis. In view of the interesting effects of resveratrol metabolites on adipocyte triacylglycerol accumulation, and in order to gain more insight into the potential involvement of resveratrol metabolites in the beneficial effects of resveratrol in obesity and insulin resistance prevention, the expression and secretion of several adipokines were analyzed in 3T3-L1 pre-adipocytes and mature adipocytes treated with three resveratrol metabolites. As far as we know, this is the first time that this issue has been addressed. For this purpose, two concentrations were used: 25 µM in maturing pre-adipocytes and 10 µM in mature adipocytes. As explained in the Materials and Methods section, the reason for using these doses was based on our previous study. Moreover, these doses are among those most commonly used in *in vitro* studies performed to analyze the effects of resveratrol on adipocytes [44]–[46].

The increase in adiponectin expression induced by resveratrol in the present study is in good accordance with the results reported by Wang *et al.* [47]. These authors showed that resveratrol increased adiponectin expression in 3T3-L1 adipocytes in a time- and dose-dependent mode. Nevertheless, other adipocytes, such as SGBS cells, do not respond to resveratrol in the same way. Derdemezis *et al.* [29] did not find any effect on adiponectin secretion when treating these cells with 10 and 25 µM of resveratrol for 24 and 48 hours on day 15 of differentiation. The reduction induced by resveratrol in leptin expression is also in line with other published results. In this context, Skudelszka *et al.* [22] observed that 62.5 µM of resveratrol reduced leptin secretion from isolated rat adipocytes.

To the best of our knowledge, very few data exist in the literature concerning the effect of resveratrol on visfatin and apelin. Only Derdemezis *et al.* [29] described that resveratrol reduced visfatin secretion in mature SGBS cells incubated with 10 and 25 µM of resveratrol for 24 and 48 hours on day 15 of differentiation. With regard to apelin, as far as we are aware, no data have been reported.

A repeated phenomenon in our study is the presence of discrepancies between mRNA and protein concentration in the culture media. Several explanations can be found in the literature to justify differences between gene and protein expression [48]–[51]: polysome activation on mRNA, alternative mRNA splicing, differences in the half-life of the protein and the mRNA, protein turnover, specific proteolytic processing. Moreover, additional factors regulate protein secretion: translocation across the plasma membrane, sequestration by secretory lysosomes, shedding of microvesicles at the extracellular side of the plasma membrane, etc [52]. In addition, several hormones have also been demonstrated to regulate protein secretion in the cell. In this context, Wang *et al.* [53] described the important effect of insulin on protein secretion but not on protein transcription. They analyzed several secreted proteins regulated by insulin, including adiponectin, and observed that although their gene expression was up-regulated, insulin inhibited their protein secretion.

Taking this into account, it could be hypothesized that in the present study insulin, although present in the culture media at a lower concentration than that used by Wang et al., could have affected protein secretion.

As mentioned in this Discussion section, in our previous study devoted to analyzing the effects of resveratrol metabolites on triacylglycerol content in the same sets of maturing pre-adipocytes and mature adipocytes [40], we observed the delipidating effect of these molecules. It is well known that adipokine expression is related to adipocyte triacylglycerol content. More specifically, lipid accumulation leads to a decrease of adiponectin expression and to a greater expression of leptin. Accordingly, in these cells the reduction observed in triacylglycerol content induced by resveratrol and resveratrol metabolites [40] was associated with the up-regulation of adiponectin and the down-regulation of leptin gene expression.

We also found [40] that the delipidating effect of resveratrol and its metabolites depended on the maturation state of the cells. Following these results, the present study was carried out in both maturing pre-adipocytes and mature adipocytes. In this regard, it is interesting to point out that in the case of adiponectin the effects of the molecules analyzed were greater in maturing pre-adipocytes than in mature adipocytes, whereas in the case of leptin the opposite was observed.

It is important to remember that in our previous study [40], resveratrol and its metabolites reduced the expression of CEBPβ, a gene involved in adipocyte differentiation, in maturing pre-adipocytes. The relationship between adipogenic genes and adipokines has been described in other studies. Mason *et al.* [54] showed that leptin expression was regulated by several transcriptional factors including CEBP, but not by PPARγ or SREBP. In the present study we analyzed the correlations between the expression of leptin and transcription factors (measured in our previous study [40]), and we found that CEBPβ and leptin were positively correlated (r = 0.593; *P*<0.001). By contrast, no significant correlation was found between PPARγ and leptin expressions. We could carry out this analysis because, although the expressions of transcription factors and adipokines are presented in two different manuscripts, they were measured in the same sets of cells. These results could suggest that the reduced expression of the transcription factor CEBPβ induced by resveratrol, and its metabolites, could have contributed to the reduction of leptin expression. Thus, the changes observed in leptin expression in maturing pre-adipocytes could be due to both direct effects of resveratrol and its metabolites on this parameter as well as to effects derived from changes in CEBPβ expression.

In summary, the present study shows for the first time the effect of resveratrol metabolites on adipokine expression and secretion in 3T3-L1 cells. Consequently, it may be suggested that both resveratrol and resveratrol metabolites are involved, to greater or lesser extents, in the regulation of energy and glycemic homeostasis of this polyphenol.
